# AURKAIP1 actuates tumor progression through stabilizing DDX5 in triple negative breast cancer

**DOI:** 10.1038/s41419-023-06115-1

**Published:** 2023-12-01

**Authors:** Wenwen Tian, Yuhui Tang, Yongzhou Luo, Jindong Xie, Shaoquan Zheng, Yutian Zou, Xiaojia Huang, Linyu Wu, Junsheng Zhang, Yuying Sun, Hailin Tang, Wei Du, Xing Li, Xiaoming Xie

**Affiliations:** 1 Sun Yat-sen University Cancer Center, State Key Laboratory of Oncology in South China, Collaborative Innovation Center for Cancer Medicine, 651 East Dongfeng Road, Guangzhou, 510060 China; 2 Affiliated Cancer Hosipital & Institute of Guangzhou Medical University, No.78 Hengzhigang Road, Guangzhou, 510095 China; 3 Breast Disease Center, The First Affiliated Hospital, Sun Yat-sen University, Guangzhou, 510080 China; 4 Department of pathology, The First People’s Hospital of Changde City, Changde, Hunan China

**Keywords:** Breast cancer, Cell migration

## Abstract

Aurora-A kinase interacting protein 1 (AURKAIP1) has been proved to take an intermediary role in cancer by functioning as a negative regulator of Aurora-A kinase. However, it remains unclear whether and how AURKAIP1 itself would directly engage in regulating malignancies. The expression levels of AURKAIP1 were detected in triple negative breast cancer (TNBC) by immunohistochemistry and western blots. The CCK8, colony formation assays and nude mouse model were conducted to determine cell proliferation whereas transwell and wound healing assays were performed to observe cell migration. The interaction of AURKAIP1 and DEAD-box helicase 5 (DDX5) were verified through co-immunoprecipitation and successively western blots. From the results, we found that AURKAIP1 was explicitly upregulated in TNBC, which was positively associated with tumor size, lymph node metastases, pathological stage and unfavorable prognosis. AURKAIP1 silencing markedly inhibited TNBC cell proliferation and migration in vitro and in vivo. AURKAIP1 directly interacted with and stabilized DDX5 protein by preventing ubiquitination and degradation, and DDX5 overexpression successfully reversed proliferation inhibition induced by knockdown of AURKAIP1. Consequently, AURKAIP1 silencing suppressed the activity of Wnt/β-catenin signaling in a DDX5-dependent manner. Our study may primarily disclose the molecular mechanism by which AURKAIP1/DDX5/β-catenin axis modulated TNBC progression, indicating that AURKAIP1 might serve as a therapeutic target as well as a TNBC-specific biomarker for prognosis.

## Introduction

Breast cancer remains the leading cause of tumor burden in global female undoubtedly [1, 2]. And approximately 10–20% breast cancers which lack of estrogen receptor, progesterone receptor (PR) and human epidermal growth factor receptor 2 (HER2) were classified into triple negative breast cancer (TNBC) and always posed therapeutic challenges for TNBC patients [3–6]. Even more unfortunate is the fact that TNBCs exhibit aggressive clinical behavior, which directly contributes to shorter survival time of this subset of breast cancer patients [7–9]. Therefore, there is still a need to explore potential specific targets for TNBC as a basis for future treatment.

AURKAIP1 (Aurora-A kinase interacting protein 1) has been identified as an Aurora-A kinase interacting protein involving in two degradation pathways of Aurora-A, including proteasome-dependent pathway and Ub-independent pathway [10–12]. Ectopic expression of AURKAIP1 resulted in the down-regulation of Aurora-A protein levels and thus, AURKAIP1 could be regarded as a cancer suppressor involving Aurora-A pathway. Interesting thing though is, elevated expression of AURKAIP1 was also observed with Aurora kinases over expressed in several types of human cancer cells derived from breast, pancreatic, and bladder cancers [13]. Apart from these contradictory findings, an even more surprising discovery was that cells overexpressing AURKAIP1 could still survive and proliferate, even though AURKAIP1 negatively regulated Aurora-A kinase which has a significant role in tumorigenesis [10, 14]. It will be full of interest to elucidate the exact role and specific mechanisms of AURKAIP1 in human cancer.

As the most studied protein of the DEAD box family of RNA helicases, DDX5 (DEAD-box helicase 5) has been confirmed to extensively participated in regulating multiple aspects of cancer development and progression [15–18]. More specifically, it is known to be a transcriptional coactivator which works together with several extremely important oncogenic transcript ion factors, such as β-catenin [19, 20], p53 [21] and nuclear factor κβ (NF-κβ) [22]. In addition to this feature, aberrant modifications of DDX5 leading to tumor progression have been observed in diverse human cancers, including breast cancer [23, 24], colon cancer [19], prostate cancer [25] and glioma [22]. Collectively, substantial studies have accentuated the indispensable role of DDX5 in achieving early diagnosis, effective treatment and predicting therapeutic response of human malignances [15].

Abnormal expression of Wnt/β-catenin signaling across human cancer is highly involved in the advancement of malignances and unfavorable prognoses of cancer patients, which facilitated increased prevalence and cancer-related mortality [26]. And with massive evidence emerging in recent decades, Wnt/β-catenin pathway has been confirmed to be strongly associated with cancer advancement and metastasis [27–29], drug resistance [30], regulation of tumor stemness [31, 32] and modulation of tumor microenvironment [33] in breast cancer.

In the current study, we revealed, for the first time, the critical function of AURKAIP1 in tumor progression of TNBC in vitro and in vivo. Strikingly, we then found that AURKAIP1 heightened the protein stability of DDX5 by directly binding to DDX5. Sequentially, AURKAIP1 promoted TNBC development in a DDX5-mediate pattern by enhancing β-catenin activity. More fundamentally, clinical relevance was evidenced by the elevation of AURKAIP1 expression in TNBC tissues and correlation with worse survival outcomes of TNBC patients. Convincingly, these promising results implicated that AURKAIP1/DDX5/β-catenin axis may be a potential target for TNBC therapy.

## Materials and methods

### Patients and samples

All primary samples of TNBC and the adjacent normal tissues were attained from surgical specimens of TNBC patients in the Sun Yat-Sen University Cancer Center (SYSUCC), and informed consents were acquired from each patient before sample collection.

### Bioinformatic analysis

First of all, application of R package DEseq2 for differential gene expression analysis between normal breast tissues and TNBC tumors was performed in Gene Expression Omnibus (GEO) database [34]. Then correlation of AURKAIP1 with clinical survival outcomes was estimated in The Cancer Genome Atlas (TCGA) database using Kaplan−Meier survival analyses and log-rank test. Survival outcomes contained overall survival (OS), recurrence-free survival (RFS), disease-specific survival (DSS) and progression-free survival (PFS), and AURKAIP1 expressions were stratified into high and low groups by median value). In addition, all public data was downloaded from the GEO database (https://www.ncbi.nlm.nih.gov/geo) and the TCGA data portal (https://tcga-data.nci.nih.gov/tcga/).

### Cell culture

Human TNBC cell lines (MDA-MB-231, BT 549, MDA-MB-468, MDA-MB-157, and HCC 1806), human normal breast epithelial cell line (MCF 10A) and the HEK 293T cell line were all purchased from American Tissue Culture Collection (ATCC, Manassas, VA, USA). Except for MCF-10A cell line cultured in DMEM/F-12 medium (Gibco, NY, USA), all other cell lines were cultured in DMEM (Gibco, USA) which contained 10% fetal bovine serum (FBS, Gibco, NY, USA) plus 1% penicillin/streptomycin (Gibco, NY, USA). All cells were regularly cultivated in a humidified incubator at 37 °C plus 5% CO^2^ (Thermo Fisher Scientific, MA, USA).

### RNA purification and quantitative real-time PCR (qRT-PCR)

Total RNA extraction (ES Science, RN001), cDNA synthesis (Takara, RR036A) and real-time qPCR (Takara, RR420A) were respectively performed following the manufacturer’s instructions. And the primers used in the study were provided in Supplementary Table 4. The relative expression of each gene was measured using the comparative threshold cycle method of calculating 2^-ΔΔCt^ [35].

### Cell transient transfection and construction of stable cell lines

It was achieved by using Lipofectamine 3000 Transfection Reagent (Invitrogen, USA) to transiently transfect siRNAs and plasmids into cells. And total RNA and protein were harvested after 48 and 72 h respectively. With screening through experimental validation, two RNA target sequences which induced significant knockdown (~70%) of AURKAIP1 were chosen for further research (siRNA#1: GCAAAAACGTGCTGAAGAT; siRNA#2: GCUGUUGAGGGCCGUUCCUTT). In MDA-MB-231 and BT 549 cells, infection with lentivirus and screening with puromycin (1 ug/ml) were conducted with standard protocol to obtain stable knockdown or overexpression cell lines. All siRNAs and plasmids were synthesized by technology companies.

### Cell proliferation and migration assays in vitro

The Cell Counting Kit-8 (CCK8) proliferation assays and colony formation assays for cell proliferation and wound healing and transwell assays for cell migration were operated in a standardized manner as previously described [36, 37]. For CCK8 assay, 1000 cells were spread in 96-well plates and cultured with normal medium, then the medium was removed and CCK8 reagent was added after adherent, 24 h, 48 h, 72 h and 96 h, and then incubated for 2 h and OD value at 450 nm was measured. For colony formation assay, 1000 cells were planted in 6-well plates and cultured for 10−14 days. After removing the medium, cells were fixed with methanol, stained with 1% crystal violet and photographed. For transwell migration assay, after transwell chamber was placed in a 24-well plate, serum-free medium containing 40,000 cells was added to the upper layer, and 20% serum medium was added to the lower layer. The medium was removed after incubating for 15−20 h, fixed with methanol, stained with 1% crystal violet, and photographed for observation. For wound healing assay, we planted the cells in 6-well plates and cultured them until the fusion reached 90%. Three vertical scratches were created in each hole and cell fragments were removed with PBS to photograph clearly. Cells were continued to grow in serum-free medium for 24 h, then the medium was removed again and photographs were taken according to the labels.

### Western blots (WB) and co-immunoprecipitation (CoIP) assay

For western blots, isolation of total proteins from indicated cells used the RIPA buffer with protease inhibitor PMSF (Beyotime, Shanghai, China), then separated in SDS-PAGE. For CoIP, protein extraction of indicated cells were achieved with IP lysis buffer containing protease inhibitor PMSF (Beyotime, Shanghai, China). Each IP condition used 80% of total protein lysate and 20% was provided as input. The IP samples mixed with primary antibodies were incubated at 4 °C overnight. Afterwards, protein A/G magnetic beads (HY-K0202, MCE) were added, incubated at 4 °C for 3 h, and washed five times. Subsequently, the beads were eluted in 1X SDS loading buffer and stored at –80 °C until processed. Primary antibodies were listed as follows: anti-AURKAIP1(PA5-50356/PA5-56869, Invitrogen), anti-DDX5 (#9877, CST), anti-Flag (66008-4-Ig/ 20543-1-AP, Proteintech), anti-HA (51064-2-AP/ 66006-2-Ig, Proteintech), anti-MYC (16286-1-AP, Proteintech), anti-β-catenin (#8480, CST), anti-Met (#8198, CST), anti-Cyclin D1 (60186-1-Ig, Proteintech), anti-c-Myc (10828-1-AP, Proteintech), anti-GAPDH (60004-1-Ig, Proteintech). And all original images were displayed in Supplementary Table 5.

### TOP/FOP luciferase reporter assay

For determining Wnt/β-catenin transcriptional activity, a TOP/FOP flash reporter assay was performed according to previous studies [38]. The HEK 293 T cells were seeded in a 24-well culture plate and transfected with TOP-FLASH or FOP-FLASH reporter plasmids together with AURKAIP1 siRNA for 48 h, then detection of luciferase activity using Dual Luciferase Reporter Gene Assay Kit (Yeasen, 11402ES60) was performed. We calculated TOP and FOP activities as follows: TOP/FOP = (top firefly luciferase activity/renila luciferase activity)/(fop firefly luciferase activity/renila luciferase activity).

### Immunofluorescence (IF) staining

Cells were incubated to 50% confluency in 24-well plates with glass coverslips at 37 °C, washed with PBS, fixed with 4% formaldehyde for 20 min, permeabilized with 0.2% Triton X-100 for 10 min, blocked with ready-to-use goat serum for 1 h at RT, and incubated with desired primary antibodies overnight at 4 °C and fluorochrome-conjugated secondary antibodies (SA00013-1/ SA00013-4, Proteintech) for 1 h at RT under dark, finally nuclei were stained with DAPI (Solarbio, China).

### Immunohistochemistry (IHC)

Briefly, tissues sections that had been paraffin-embedded were dewaxed with xylene and rehydrated with gradient ethanol after baking at 65 °C for 2 h. In order to retrieve antigens, the slides were immersed in sodium citrate (pH 6.0) for 30 min and steamed. Preliminary antigen retrieval was performed using pH 9.5 EDTA antigen retrieval buffer through boiling for 10 min with a pressure cooker. After natural cooling to room temperature, the peroxidase blocker was added to the sections for 10 min to quench the internal peroxidase activity. Then the slides were washed three times with PBS, blocked with normal goat serum at 37 °C for 30 min and incubated with the primary antibody (anti-AURKAIP1, PA5-50356, Invitrogen) at 4 °C overnight. The next day, the slides were cleaned by PBS for three times, incubated in homologous secondary antibody for 1 h at room temperature, another three times washing, visualized using DAB peroxidase substrate kit, counterstained using haematoxylin and dehydrating in graded ethanol. Finally, the sections were baked at 65 °C for 5 min and sealed before quantitative analysis. Unless otherwise stated, all reagents were supplied from ZSBIO (Beijing, China). Semiquantitative analyses was performed by multiplying the percentage of positive-staining cells (scored 0 to 4) by the staining intensity (ranked 1 to 3) as previously reported [39].

### Protein stability detection

The cycloheximide (CHX)and proteasome inhibitor (MG132) treatment were used for assessing the protein stability of DDX5. For CHX, indicated cells transfected with scramble or AURKAIP1 siRNAs were processed with CHX (50 μg/ml) and collected at the appointed time points. Moreover, we treated TNBC cells with MG132 (50uM) for 6 h after AURKAIP1 or scramble siRNA transfection and harvested the total proteins. The CHX and MG132 were both purchased from Yeasen (Guangzhou, China).

### In vitro ubiquitination assay

The HEK 293T cells were co-transfected with MYC-Ub, HA-DDX5 and Flag-AURKAIP1 or shAURKAIP1 plasmids. After 48 h, cells were subsequently treated with MG132 (25 uM) for another 6 h before collected with IP lysis buffer. The rest of the steps were carried out according to the protocol of IP and further verified by SDS–PAGE for immunoblotting.

### TNBC xenograft tumor model in vivo

In total, twenty BALB/c nude mice (female, 3–4 weeks old) were purchased from the Beijing Vital River Laboratory and divided into four groups (*n* = 5 for each group) randomly for subsequent experiments. 5 × 10^6^ cells of each stable TNBC cell lines (shScramble-MDA-MB-231, shAURKAIP1-MDA-MB-231, shScramble-BT 549, shAURKAIP1-BT 549) were suspended with 100 ul PBS and then injected into the unilateral mammary fat pads of every nude mouse. The tumors were measured and recorded every 5 days until harvested from sacrificed mice after 3 weeks of inoculation. Calculation of tumor volume: (length × width^2^)/2. The performance of animal experiments in vivo fulfilled the principles for the use of laboratory animals of the Sun Yat-Sen University Institutional Animal Care and Use Committee.

### Statistical analysis

The results were expressed with mean ± SD and statistical analyses were conducted by one-way ANOVA, two-way ANOVA or Student’s t-test analysis using GraphPad Prism software unless otherwise stated. Spearman test was used to examine correlations. *p* < 0.05 was considered statistically significant and denoted by asterisks (*p < 0.05, **p < 0.01, ***p < 0.001 and ****p < 0.0001). All experiments in vitro were repeated for at least three independent times.

## Results

### AURKAIP1 was upregulated in TNBC and correlated with unfavorable clinical outcome

**Fig. 1:**
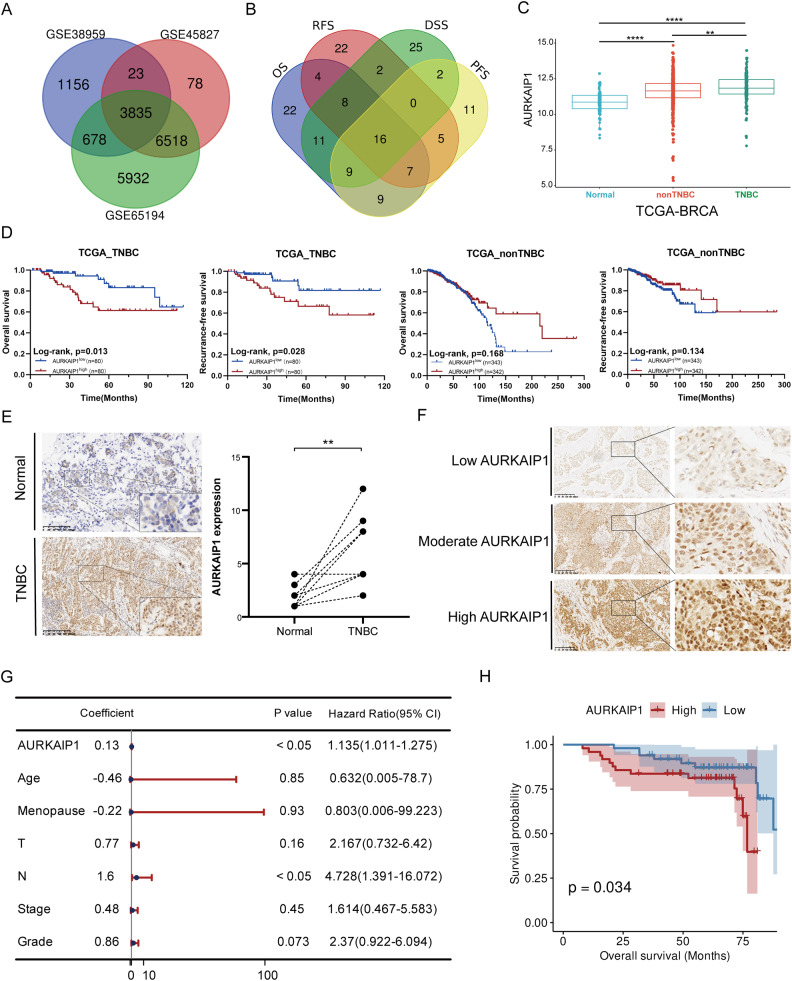
Upregulated AURKAIP1 expression was observed in triple-negative breast cancer (TNBC) and correlated with worse prognosis in patients with TNBC. **A** The Venn diagram displayed the common differentially expressed genes (DEGs) between normal and TNBC samples in multiple GEO datasets (GSE38959, GSE45827 and GSE65194). **B** Survival analyses of overall survival (OS), recurrence-free survival (RFS), disease-specific survival (DSS) and progression-free survival (PFS) for obtained DEGs in TCGA-BRCA. **C** Comparison of AURKAIP1 mRNA expression levels in TCGA-BRCA. **D** Survival analyses of OS and RFS in non-TNBC and TNBC patients of TCGA-BRCA based on different AURKAIP1 expression levels. **E** The detection of AURKAIP1 expression in TNBC and para-cancer tissues from 10 TNBC patients by IHC. **F** Immunostaining of a tissue microarray consisting of 100 human samples with TNBC for AURKAIP1. **G** Multivariate Cox regression analysis for AURKAIP1 expression and clinicopathological features to determine whether AURKAIP1 could be recognized as an independently prognostic indicator for OS of TNBC patients. **H** Kaplan–Meier survival analysis of the association of AURKAIP1 protein levels with OS (*n* = 100, *p* = 0.034) in 100 patients with TNBC.

To enable better understanding of the TNBC molecular basis and to search of candidate biomarkers for clinical application, we firstly screened the differentially expressed genes (DEGs) between TNBC tissues and normal breast samples using multiple GEO datasets (GSE38959, GSE45827 and GSE65194) and obtained 3835 DEGs (|log2FC| >1 and FDR < 0.05) in TNBC samples comparing with normal tissues (Fig. 1A, Supplementary Table 1). To further narrow the DEGs, we performed Kaplan–Meier survival analyses of 3835 DEGs in the TCGA-BRCA dataset to find prognostic genes (Log rank test *p* < 0.05) associated with OS, RFS, DSS and PFS in TNBC patients (Supplementary Table 2). Given these initial results, 16 candidate genes were considered to gain more insight (Fig. 1B). After summarizing the current analysis results and the reported literatures, we finally determined AURKAIP1 for exploring its role in tumor progression of TNBC.

To inquire into the prognostic value and expression of AURKAIP1, TCGA-BRCA data was analyzed first, and the result showed that AURKAIP1 was manifestly upregulated in TNBC samples comparing with unpaired normal breast tissues and non-TNBC samples (Fig. 1C). Meanwhile, by assessing the survival probability between different AURKAIP1 expression, we strikingly found that high AURKAIP1 expression was associated with poorer OS and RFS in TNBC patients rather than non-TNBC patients, which patently evidenced that AURKAIP1 may be a TNBC-specific prognostic biomarker (Fig. 1D). Further proving these findings, high AURKAIP1 expression was validated through IHC in cancerous and matched adjacent noncancerous tissues of 10 TNBC patients from SYSUCC (Fig. 1E). Then a tissue microarray (TMA) containing 100 TNBC samples was used to verify the association of AURKAIP1 expression with age, menopause status, tumor grade, T stage, N stage and TNM stage (Fig. 1F). The result revealed that AURKAIP1 expression was dramatically correlated with T stage, N stage and stage of TNBC patients (Table 1). In particular, TNBC patients with high AURKAIP1 expression tended to have larger tumor, more axillary lymph node metastasis and severer clinical stage. Further, multivariate Cox regression analysis implied that AURKAIP1, as well as N stage, might have the potency to work as an independently prognostic indicator for TNBC (Fig. 1G). Moreover, TNBC patients with higher AURKAIP1 levels tended to have worse OS probability from the Kaplan–Meier survival analysis (Fig. 1H), which reiterated that AURKAIP1 was a detrimental feature of TNBC.

**Table 1:** The association between AURKAIP1 expression and clinicopathological factors in TNBC patients (*n* = 100).

Parameters		AURKAIP1 expression		*p* value
		High (*n* = 50)	Low (*n* = 50)	
Age	≤50	27	35	ns
	>50	23	15	
Menopause	Yes	22	15	ns
	No	28	35	
Grade	I + II	23	26	ns
	III	27	24	
T	T1-T2	39	49	**
	T3-T4	11	1	
N	Negative	15	39	****
	Positive	35	11	
Stage	I + II	26	42	****
	III + IV	24	8	

Chi-square test was used for comparing high and low groups of AURKAIP1 expression, *p < 0.05 was considered significant; ns, not significant; **p < 0.01; ****p < 0.0001.

### Altered AURKAIP1 expression impacted TNBC cell proliferation and migration in vitro and in vivo

**Fig. 2:**
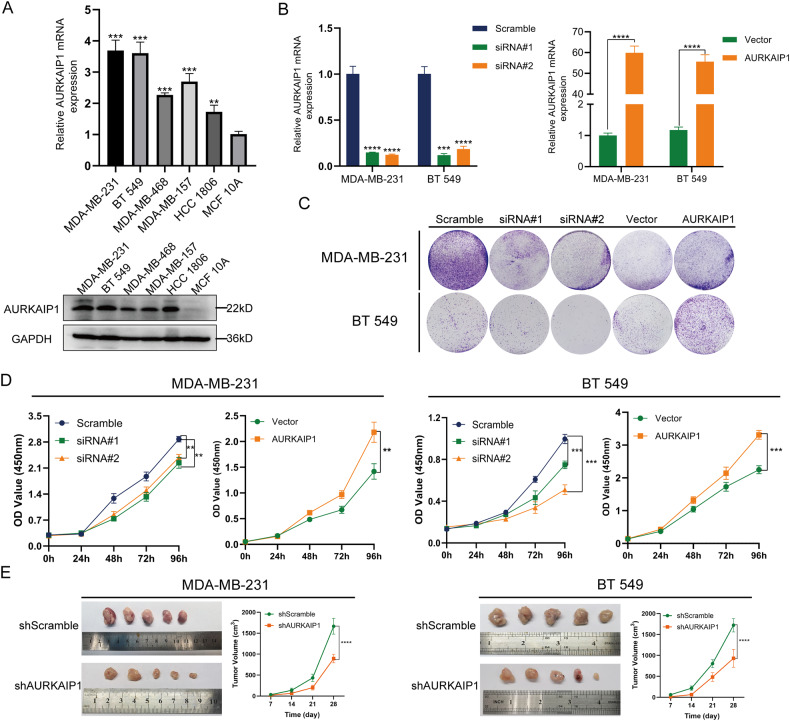
AURKAIP1 promoted cell proliferation and migration of triple-negative breast cancer (TNBC). **A** The relative mRNA and protein expression levels of AURKAIP1 in normal breast cell (MCF 10A) and five TNBC cell lines. **B** RT-qPCR analyses of mRNA expression level in the TNBC cells after knockdown or overexpression of AURKAIP1. **C** The effects induced by AURKAIP1 suppression and overexpression on colony-forming ability were detected by colony formation assays. **D** The CCK8 assays were performed to evaluate the cell viability of TNBC cells transfected with AURKAIP1 siRNAs or plasmids. **E** Images and tumor growth curves of subcutaneous tumor formation in nude mice with stable shScramble or shAURKAIP1 cells (*n* = 5 for each group). Data are represented with mean ± SD from three independent experiments (*p < 0.05; **p < 0.01; ***p < 0.001, ****p < 0.0001).

**Fig. 3:**
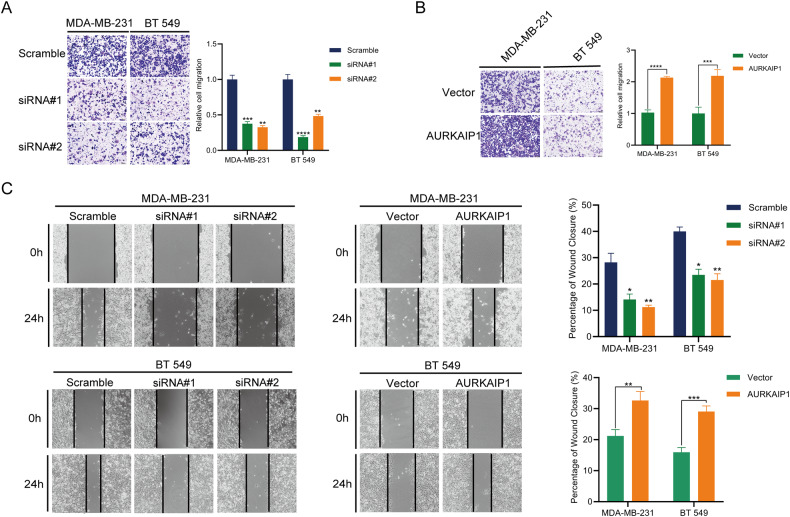
AURKAIP1 contributed to cell migration in TNBC. Representative images and quantified data of the transwell (**A**, **B**) and wound healing (**C**) migration assays were presented. Data are represented with mean ± SD from three independent experiments (*p < 0.05; **p < 0.01; ***p < 0.001, ****p < 0.0001).

By qRT-PCR and western blots, we determined the mRNA and protein levels of AURKAIP1 in various cell lines, which indicated that AURKAIP1 expression was significantly higher in TNBC cells comparing with normal breast cell line MCF 10A (Fig. 2A). To establish the biological effect of AURKAIP1 on TNBC, we firstly knocked down or overexpressed AURKAIP1 in both MDA-MB-231 and BT 549 cell lines (Fig. 2B). The CCK8 proliferation experiment (Fig. 2D) and clone formation assays (Fig. 2C) showed that the proliferation capacity of TNBC cells was greatly inhibited by siRNA-based AURKAIP1 knockdown while completely enhanced by overexpression of AURKAIP1. Importantly, these expectations in vitro were further confirmed with xenograft experiments in vivo (Fig. 2E). Then, we performed transwell (Fig. 3A, B)) and wound healing assays (Fig. 3C) to assess whether AURKAIP1 affected TNBC cell migration. The results demonstrated that AURKAIP1 silencing reduced cell migration ability exceptionally, while AURKAIP1 overexpression showed the opposite effect. Taken together, AURKAIP1 inactivation impaired cell proliferation and migration of TNBC in vitro and in vivo.

### AURKAIP1 boosted TNBC advancement in a DDX5-mediated mode

**Fig. 4:**
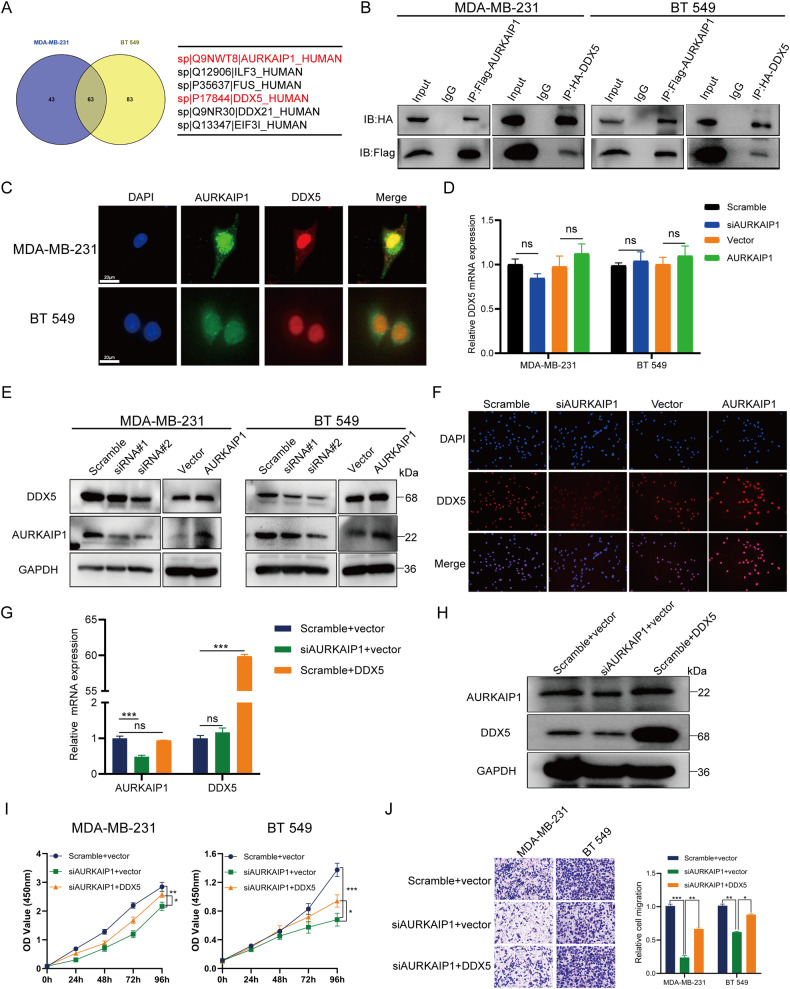
AURKAIP1 interacted with DDX5 directly and facilitated TNBC advancement though the mediation of DDX5. **A** The result of anti-AURKAIP1 immunoprecipitation followed by mass spectrometry (IP-MS) in MDA-MB-231 and BT 549 cells identified DDX5 as an interacting partner of AURKAIP1. **B** Co-IP assay were conducted to confirm the direct interaction of AURKAIP1 and DDX5. **C** Representative images of immunofluorescence assays to identify the protein localization of both AURKAIP1 and DDX5. (**D**–**F**) The expression of DDX5 was detected by western blots (**D**), RT-qPCR (**E**) and immunofluorescence assay (**F**) after indicated alteration of AURKAIP1 in TNBC cells. RT-qPCR (**G**) and western blots (**H**) were used to examine that whether DDX5 overexpression have an effect on AURKAIP1 expression in the indicated cells. The DDX5-rescued CCK8 (**I**) and transwell migration (**J**) assays verified that upregulated AURKAIP1 induced TNBC proliferation and migration via DDX5 mediation. Data are represented with mean ± SD from three independent experiments (*p < 0.05; **p < 0.01; ***p < 0.001, ****p < 0.0001).

In order to discover the mechanisms regarding how AURKAIP1 promoted TNBC progression, a co-immunoprecipitation (CoIP) and tandem mass spectrometry (MS) approach was applied to survey AURKAIP1-interacting proteins. Combing the CoIP-MS data of both MDA-MB-231 and BT 549 cells after excluding data of negative control with IgG, 63 proteins were identified to have interaction with AURKAIP1 (Fig. 4A, Supplementary Table 3). Since DDX5 has been elucidated to have a well-defined mechanism of action in tumors including breast cancer by reviewing the literature, we established DDX5 as a reciprocal target and further examined whether AURKAIP1 fostered TNBC development via the DDX5 mediator. As expected, further CoIP tests in both MDA-MB-231 and BT 549 cells supported the direct binding of DDX5 to AURKAIP1 (Fig. 4B). Moreover, co-localization of the AURKAIP1 protein with DDX5 protein was also revealed by double immunofluorescent staining (Fig. 4C). Taken together, the current experimental evidence indicated that DDX5 and AURKAIP1 directly interacted with each other. Subsequently, we investigated the specific regulatory effects of AURKAIP1 on DDX5. From the result, we found that AURKAIP1 reduction clearly decreased DDX5 protein levels (Fig. 4E), while mRNA levels had no visible changes (Fig. 4D). Correspondingly, overexpression of AURKAIP1 equally increased the expression of DDX5 protein level rather than the mRNA standard. This observation was reiterated by cellular immunofluorescent (Fig. 4F), which clarified the underlying mechanism that AURKAIP1 may be engaged in DDX5 modulation through a post-translational manner.

Given that AURKAIP1 had a regulatory relationship with DDX5, it was worthwhile to examine whether AURKAIP1 acted through DDX5 to exert its oncological effects on TNBC tumors. Both qRT-PCR and western blots experiments indicated that overexpressing DDX5 purely in MDA-MB-231 cells could not alter AURKAIP1 expression, which provided a clue for DDX5 as a downstream molecule of AURKAIP1 (Fig. 4G, H). Even more convincing was the fact that DDX5 overexpression in AURKAIP1 knockdown cells could comparably reversed the oncogenic phenotype produced by silencing AURKAIP1 in CCK8 (Fig. 4I) and transwell migration assays (Fig. 4J). Hence, it was indeed DDX5 that mediated the function of AURKAIP1 in TNBC development.

### AURKAIP1 silencing contributed to ubiquitin-degradation of DDX5

**Fig. 5:**
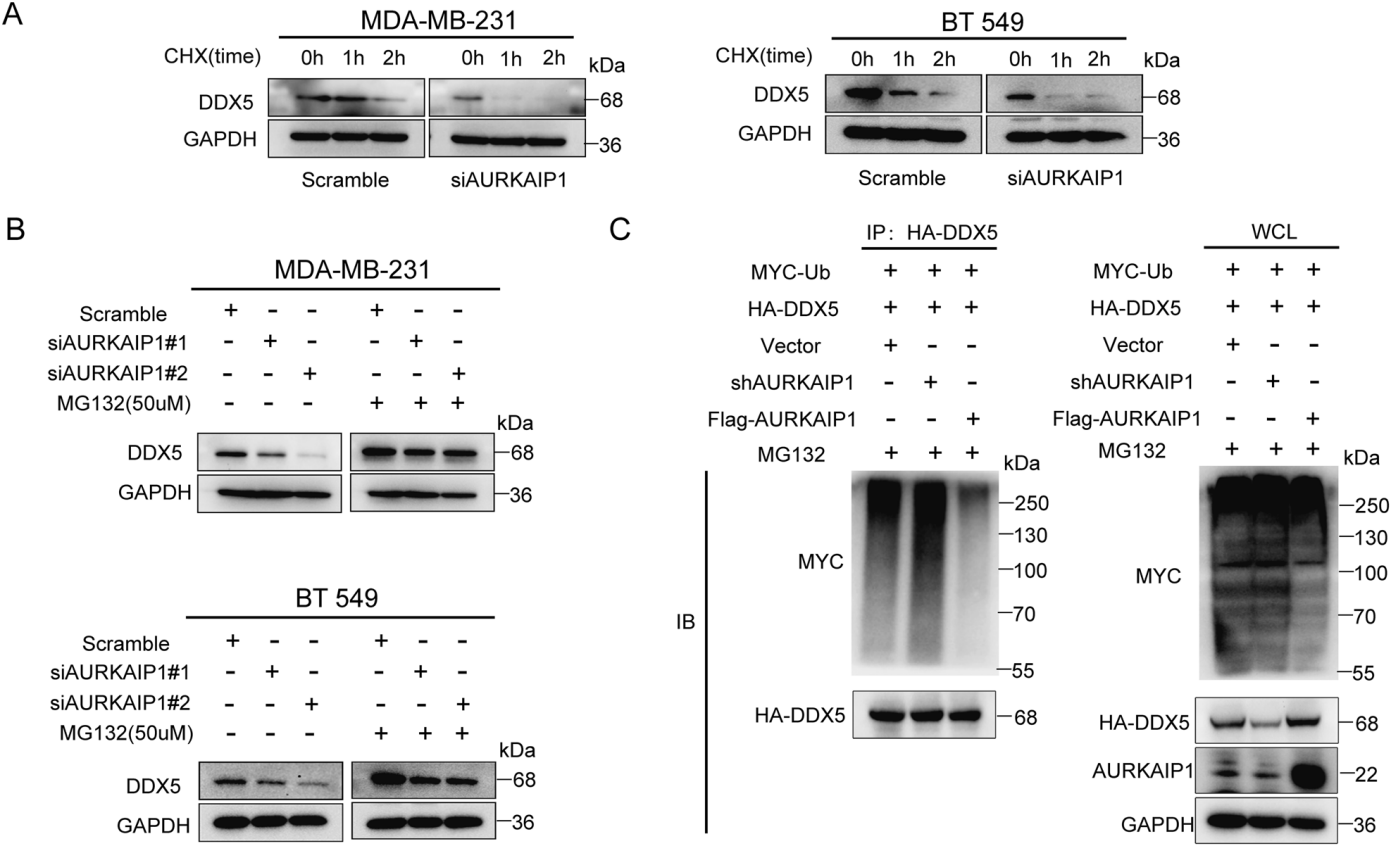
AURKAIP1 stabilized DDX5 by blocking ubiquitin-degradation of DDX5. **A** TNBC cells transfected with scramble or AURKAIP1 siRNAs were treated with cycloheximide (CHX) for indicated time periods and protein stability of DDX5 was measured by WB. **B** The protein expression of DDX5 was detected by western blots in TNBC cells transfected with scramble or AURKAIP1 siRNAs for 48 h and treated with proteasome inhibitor MG132 (50 μM) for 6 h. **C** AURKAIP1 reduced DDX5 ubiquitination. The HEK 293T cells were transfected with HA-DDX5, MYC-Ub and Flag-AURKAUP1 or shAURKAIP1 plasmids and treated with 25 μM MG132 for 6 h. Immunoprecipitation and immunoblotting were performed using the selected antibodies.

Naturally, we next investigated how deactivation of AURKAIP1 diminished the DDX5 protein. Since the impact of AURKAIP1 on DDX5 lied at only the protein level, we speculated that AURKAIP1 may modulate DDX5 protein by degradation based on the above results. To validate this surmise, we blocked protein synthesis using CHX (50 ug/ml) and detected a shorter half-life of DDX5 in AURKAIP1-knockdown MDA-MB-231 and BT 549 cells (Fig. 5A). Intriguingly, the proteasome inhibitor MG132 recovered the decreased abundance of DDX5 protein elicited by AURKAIP1 silencing (Fig. 5B). It was further shown that AURKAIP1 maintained the protein stability of DDX5 to mitigate its degradation.

Although post-translational modifications of DDX5 encompassed ubiquitination and phosphorylation as well as sumorylation [18, 40, 41], the above results suggested that AURKAIP1 affected DDX5 proteolysis through ubiquitination. To further confirm this, MYC-Ub and HA-DDX5 plasmids were co-transfected in the HEK 293T cells which were transfected with AURKAIP1 shRNA or Flag-AURKAIP1 plasmids concurrently. And the detection of DDX5-ubiquitin showed that HA-DDX5 ubiquitination was markedly augmented when transfected with siRNA of AURKAIP1 over the blank control (Fig. 5C). Meanwhile, we noticed that HA-DDX5 ubiquitination was relatively reduced when transfected with Flag-AURKAIP1. Taken together, these results implied that AURKAIP1 attenuated DDX5 ubiquitination and consequently curtailed its proteasomal degradation.

### AURKAIP1 triggered the Wnt/β-catenin signaling pathway via targeting DDX5

**Fig. 6:**
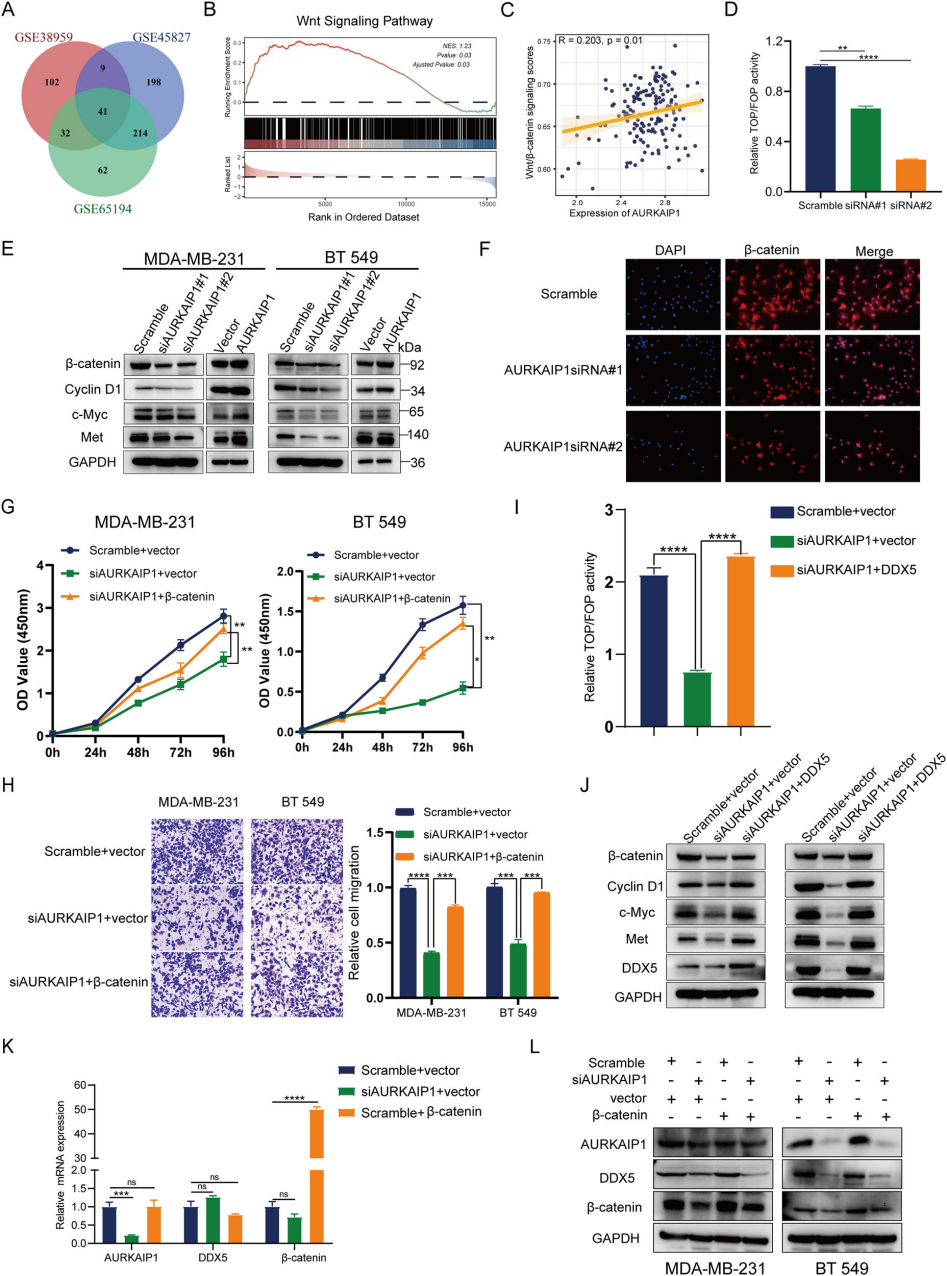
AURKAIP1 activated Wnt/β-catenin signaling pathway through targeting DDX5. **A** Veen diagram was used to present the intersection results of Gene Set Enrichment Analysis (GSEA) in different GEO datasets. **B** Representative plots of GSEA results. **C** Scatter plot showing the expression correlation between AURKAIP1 and Wnt/β-catenin signaling scores. **D** Assays of TOP/FOP luciferase activity was conducted to examine the Wnt activity in the HEK 293T cells transfecting with scramble or AURKAIP1 siRNAs. **E** Western blot analysis of β-catenin, CyclinD1, c-Myc and Met in indicated cells. **F** Immunofluorescence analysis of β-catenin in MDA-MB-231 cells transfected with scramble or AURKAIP1 siRNAs. The β-catenin rescued CCK8 (**G**) and transwell migration (**H**) assays revealed that AURKAIP1 promoted TNBC proliferation and migration via Wnt/β-catenin signaling pathway. **I** TOP/FOP flash reporter assay in indicated HEK 293T cells was performed to reaffirm that DDX5 overexpression could reverse the reduction of Wnt/β-catenin transcriptional activity induced by AURKAIP1 knockdown. **J** Western blot was used to detect the protein levels of β-catenin, CyclinD1, c-Myc and Met in indicated cells to verify whether DDX5 overexpression could invert the effect of AURKAIP1 silencing on essential proteins of Wnt/β-catenin signaling pathway. RT-qPCR (**K**) and western blots (**L**) analyses were performed to investigate the upstream-downstream relationship between AURKAIP1, DDX5 and β-catenin in the indicated cells.

Ample research findings in recent decades have shown that DDX5 could form a complex with β-catenin protein, which stimulated β-catenin transcriptional capacity and thus triggered the transcription of downstream target genes in human cancers [20, 42–44]. Meanwhile, we performed gene set enrichment analysis (GSEA) in source datasets (GSE38959, GSE45827 and GSE65194) to unravel the signaling pathways involved in AURKAIP1 function (Fig. 6A). From the result, AURKAIP1 function was strongly linked to Wnt/β-catenin signaling pathway (Fig. 6B). Moreover, the expression data of AURKAIP1 and Wnt/β-catenin signaling pathway displayed a significantly positive correlation in TCGA-TNBC (Fig. 6C). In relying on these corroborating research evidence, we speculated that AURKAIP1 functioned in TNBC through DDX5-mediated regulation of Wnt/β-catenin signaling pathway. First, the TOP/FOP-flash luciferase assay also revealed that AURKAIP1 silencing could depressed the Wnt/β-catenin signaling clearly (Fig. 6D). To confirm that AURKAIP1 indeed has an effect on Wnt/β-catenin signaling, the protein levels of β-catenin, CyclinD1, c-Myc and Met were detected by WB analyses (Fig. 6E). As expected, the β-catenin and its target genes were suppressed simultaneously at the protein level by knockdown of AURKAIP1 and similarly elevated by overexpressing AURKAIP1. In addition, the protein change of β-catenin was also visualized by cellular immunofluorescence (Fig. 6F). The rescue experiments of CCK8 and transwell migration assays again reflected that AURKAIP1 did have a positive effect on TNBC by regulating Wnt/β-catenin signaling pathway (Fig. 6G, H).

**Fig. 7:**
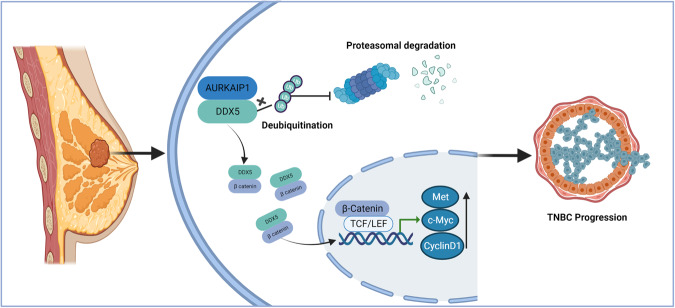
The visual presentation of the AURKAIP1-DDX5-β-catenin axis in TNBC progression.

The focus of our subsequent experiments was to verify whether AURKAIP1 participated in the Wnt/β-catenin signaling pathway by means of DDX5 mediation. Overexpression of DDX5 notably improved the reduction of TOP/FOP-flash luciferase activity caused by AURKAIP1 silencing (Fig. 6I). Equally, the protein downregulation of selected key molecules involving in Wnt/β-catenin pathway which resulting from AURKAIP1 knockdown was in turn effectively reversed by DDX5 overexpression (Fig. 6J). Additionally, we tested the upstream-downstream relationship between AURKAIP1, DDX5 and β-catenin, which showed that AURKAIP1 could function upstream of β-catenin as a positive regulator (Fig. 6K–M). Ultimately, the mechanism of AURKAIP1 function in TNBC was illustrated in Fig. 7 visibly. Up to this point, these findings indicated that AURKAIP1 might be capable of a novel tumor-promoting role in TNBC, which suggested for the first time that AURKAIP1 did have a distinct role in human cancer which did not rely upon the Aurora-A regulation.

## Discussion

Along with the evolution of high-throughput sequencing technology as well as bioinformatics analyses, considerable progress has been achieved in understanding the TNBC biology [45–47]. However, with the emergence of therapy bottlenecks in TNBC patients, it is growing more urgent and critical to identify reliable prognostic biomarkers which could be applied to distinguish clinical phenotypes and predict patient outcomes to ensure more effective clinical care. In this study, we made use of public information from GEO database to extract genes which abnormally expressed in TNBC compared to normal breast samples. And the Kaplan–Meier survival analyses of TCGA data were performed to identify prognostic genes for TNBC among DEGs. Finally, AURKAIP1 was chosen for more in-depth research in view of its apparently high expression and the specificity of prognostic relevance in TNBC.

AURKAIP1 is a generally expressed nuclear protein which reacted specifically with human Aurora-A in vivo [10]. The primary identification of AURKAIP1 was due to it targeting Aurora-A for degradation with a proteasome-dependent approach. In fact, Overexpression of Aurora-A has been proven to be dramatically related to poor prognosis and tumorigenesis of human primary tumors [48–50]. Though the above reports obliquely implied that AURKAIP1 might exert cancer-suppressive effect through negatively regulation of Aurora-A, there was no tangible evidence to elucidate the clear mechanism of its behavior in cancer so far. In this study, we have firstly confirmed that AURKAIP1 was truly upregulated in tissues and cell lines of TNBC, which was consistent with the previously reported clues [13]. Next, the correlation between AURKAIP1 expression and survival times of TNBC patients were estimated by bioinformatics analyses and IHC. And the observation suggested a strong association between highly AURKAIP1 expression and inferior prognosis in TNBC, which seems to prompt us that AURKAIP1 is very likely to be a cancer-promoting partner. To confirm the reliability of this initial observation, we silenced AURKAIP1 by targeting mRNA with siRNA in TNBC cell lines and tracked alterations in cell proliferation and migration. Not surprisingly, downregulation of AURKAIP1 obviously lead to a pro-oncogenic state, which thoroughly established AURKAIP1 as a cancer-promoting factor. To this point, we suggested that AURKAIP1 might play a predominantly pro-carcinogenic role, regardless of being a secondary regulator to suppress cancer.

By CoIP-MS, DDX5 was ascertained in the AURKAIP1-protein complex and further confirmed by immunoprecipitation and western blotting assays. High levels of DDX5 have been reported in breast cancer previously and DDX5 has been considered as a hopeful therapeutic candidate for DDX5-amplified breast tumors [17, 51]. Our findings indicated that positive modulatory response of DDX5 by AURKAIP1 facilitated the progression of TNBC, which reinforced the role of AURKAIP1 in cancer.

Since DDX5 has been researched to be involved in Wnt/β-catenin pathway by protecting β-catenin from degradation in the cytoplasm or by enhancing the transcriptional activity of β-catenin in the nucleus, we posited an AURKAIP1/DDX5/β-catenin axis and performed validation. Notably, we noticed that only DDX5 but not β-catenin was recognized in the AURKAIP1 protein complex, although DDX5 was found to interplay with β-catenin physically [42]. The result suggested that AURKAIP1-DDX5 complex and DDX5-β-catenin complex were independently active and spatially separated. As for the interaction between AURKAIP1 and DDX5, our evidence demonstrated that DDX5 ubiquitination level was responsive to proteasome inhibitor MG132 and AURKAIP1 inhibited DDX5 ubiquitination and proteasome degradation in TNBC cells, while the involved ubiquitination sites need to be further investigated.

Another very impressive finding in this study was that knockdown of AURKAIP1 using siRNA have no effects on the mRNA level of β-catenin, which might reflect that AURKAIP1 functioned in the cytoplasm through DDX5-mediated stabilization of β-catenin protein, rather than directly affecting the transcriptional activity of β-catenin. Moreover, we determined that AURKAIP1 achieved its role in TNBC by protecting β-catenin from degradation through DDX5 in the cytoplasm. This procedure plausibly explained that the cancer-suppressive effect caused by AURKAIP1 knockdown could be effectively rescued by overexpression of both DDX5 and β-catenin respectively.

It is worth mentioning that TNBC is heterogeneous, which manifested not only in histology, but also at transcriptomic level [52]. TNBC was identified several clusters, including basal-like 1 (BL1), basal-like 2 (BL2), immunomodulatory (IM), mesenchymal (M), mesenchymal stem-like (MSL), luminal androgen receptor (LAR), and unstable (UNS) [53]. And patients with TNBC always have a poor prognosis due to high inter-tumor heterogeneity [54]. Moreover, TNBC became drug resistant during treatment due to significant intra-tumor cell heterogeneity [55]. Overall, it’s necessary to perform more exploration for investigating which cluster that AURKAIP1/DDX5/β-catenin axis mainly focus on.

In conclusion, we primarily proved that AURKAIP1 was upregulated and linked to poor prognosis in TNBC. AURKAIP1 could directly interact with and stabilize DDX5 protein, raising its expression and β-catenin activity. Treatment of AURKAIP1 with siRNA reduced tumor growth, which portended that AURKAIP1 was essential for tumorigenesis and aggressiveness of TNBC. Owing to the limitations caused by specimens, the association of AURKAIP1 with DDX5 or β-catenin amplification levels need further investigation with more TNBC cases. Now that AURKAIP1 could play a non-negligible role in TNBC, its additional protein chaperones and possible mechanisms in human cancer deserved to be pursued at length.

### Supplementary information

Original data file

Suplementary Table 1

Suplementary Table 2

Suplementary Table 3

Suplementary Table 4

Reproducibility checklist

## Data Availability

The data and methods in this article would be available from the corresponding author with reasonable requirement.
