# Supplementary material for: AURKAIP1 actuates tumor progression through stabilizing DDX5 in triple negative breast cancer
Source: Cell Death Dis. 2023 Dec 1;14(12):790. doi: 10.1038/s41419-023-06115-1 (PMC10692340; doi:10.1038/s41419-023-06115-1)

Figure 2

Figure 2A

AURKAIP1

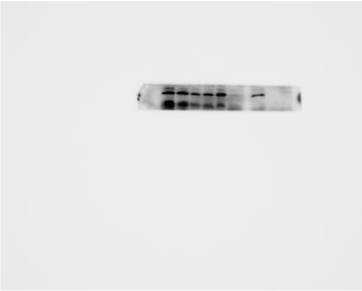

GAPDH

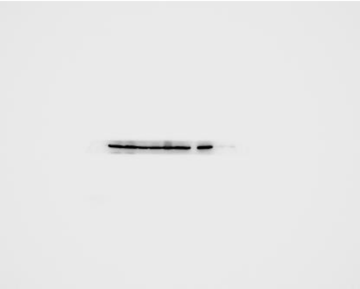

Figure 4

Figure 4B

FlagIP-Flag (left: MDA-MB-231; right: BT549)

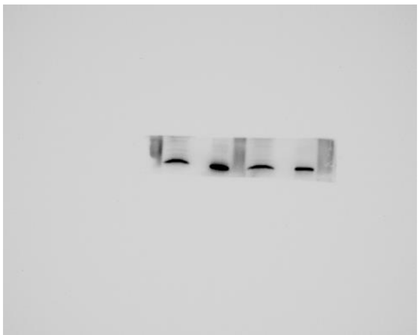

HAIP-HA (left: MDA-MB-231; right: BT549)

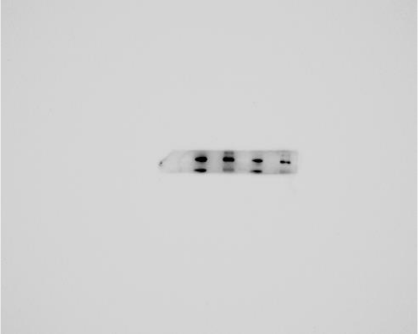

FlagIP-HA (MDA-MB-231)

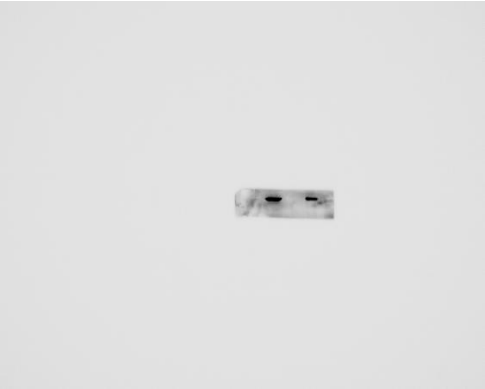

FlagIP-HA (BT549)

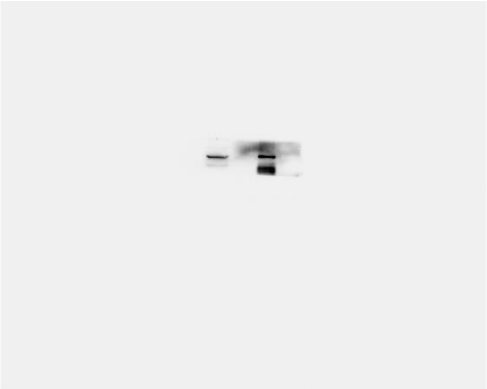

HAIP-Flag (MDA-MB-231)

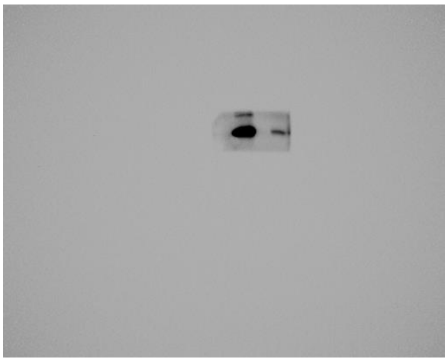

HAIP-Flag (BT549)

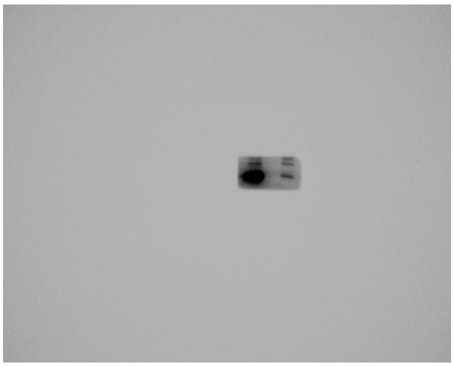

Figure 4E (MDA-MB-231)

AURKAIP1

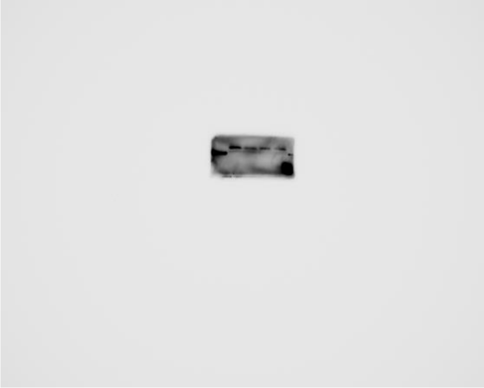

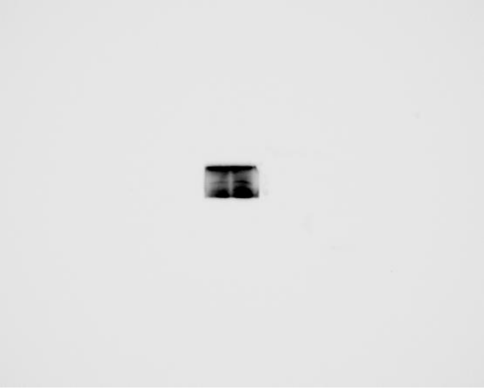

DDX5

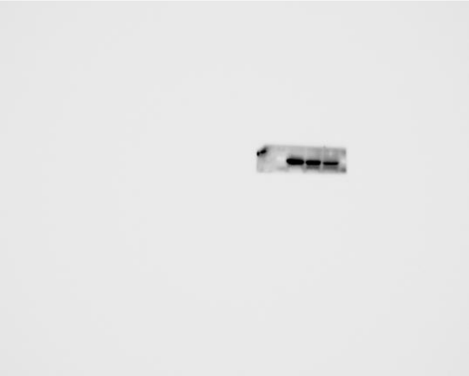

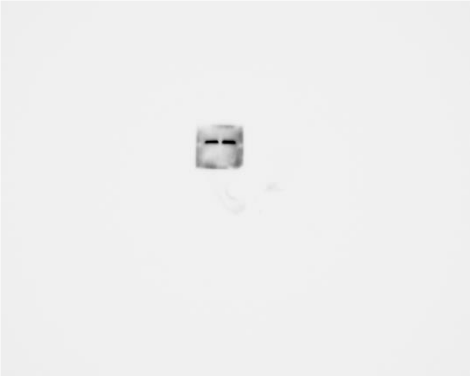

Figure 4E (BT549)

AURKAIP1

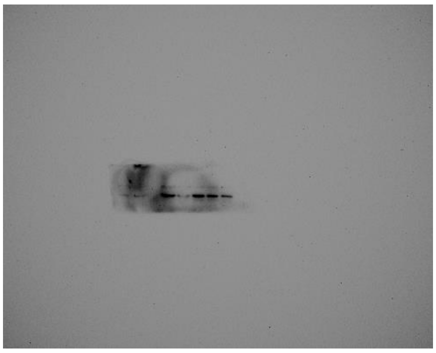

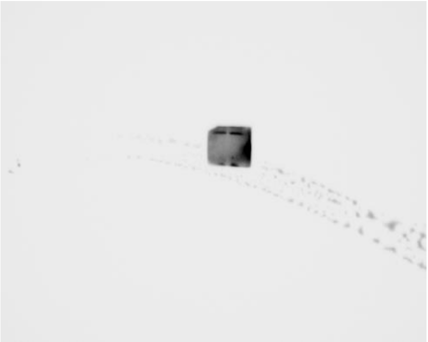

DDX5

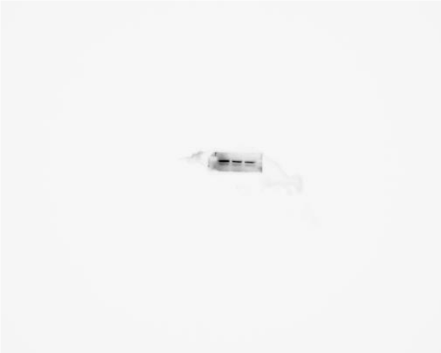

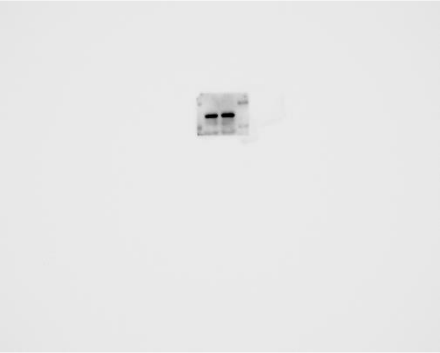

GAPDH

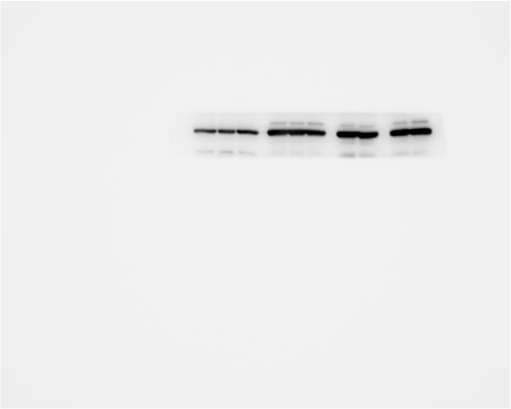

Figure 4J

AURKAIP1 DDX5 GAPDH

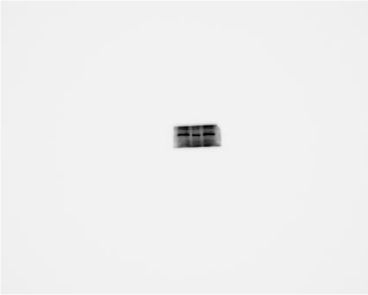

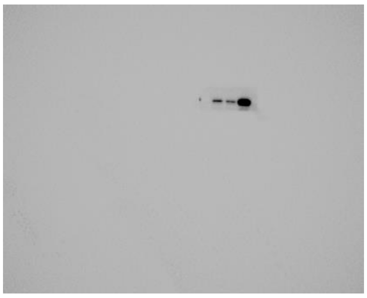

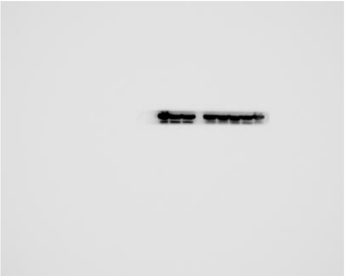

Figure 5

Figure 5A (MDA-MB-231)

DDX5 GAPDH

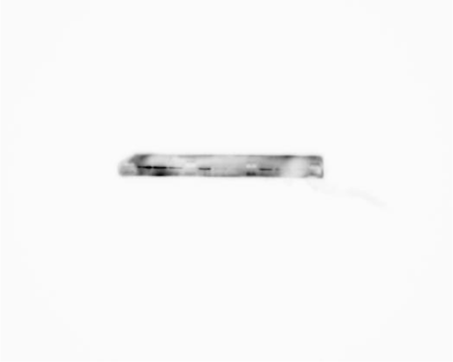

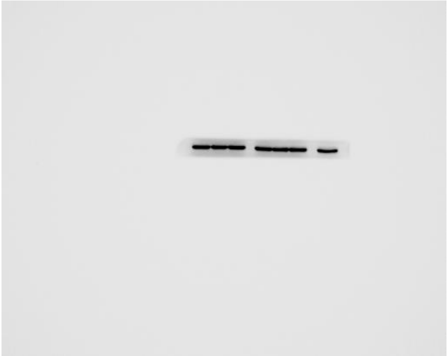

Figure 5A (BT 549)

DDX5 GAPDH

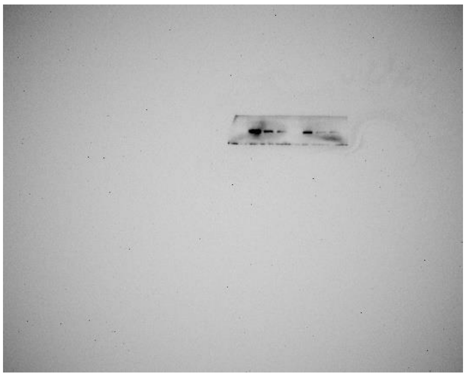

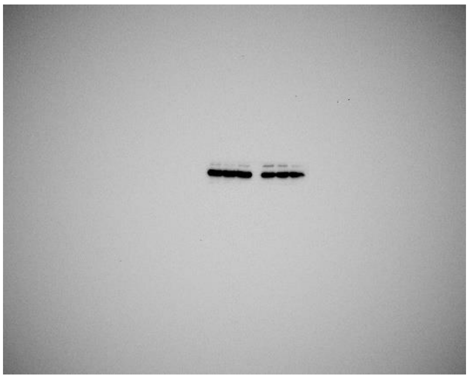

Figure 5B (MDA-MB-231)

DDX5 GAPDH

 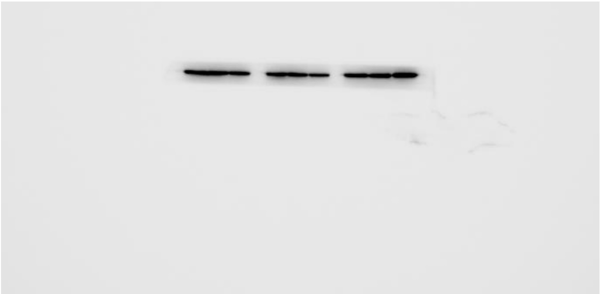

Figure 5B (BT 549)

DDX5 GAPDH

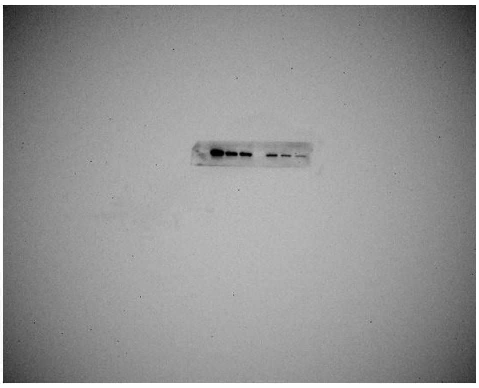 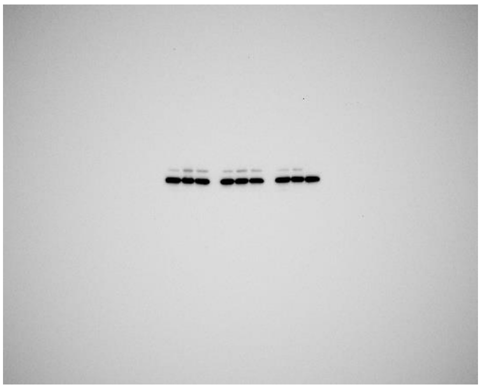

Figure 5C

Figure 5C (IP: HA-DDX5)

MYC HA-DDX5

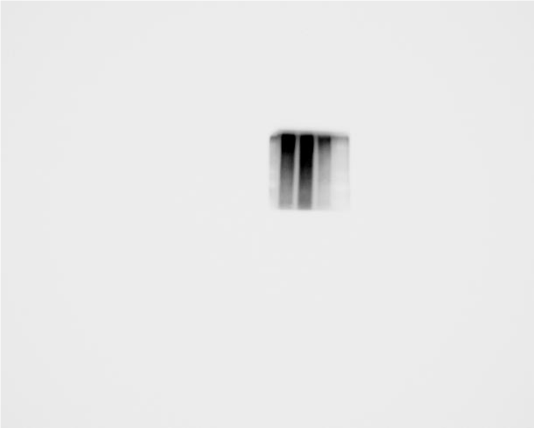 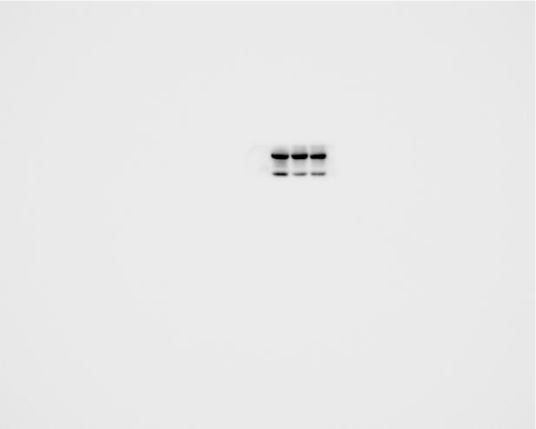

Figure 5C (WCL)

MYC

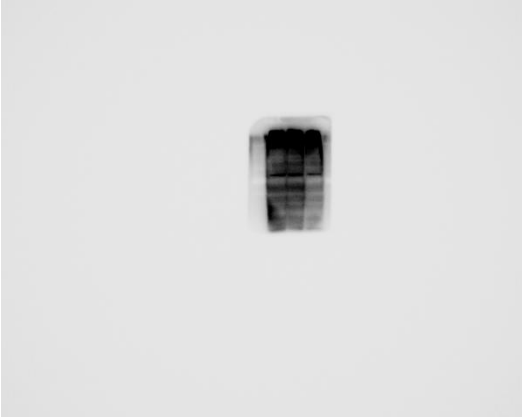

HA-DDX5

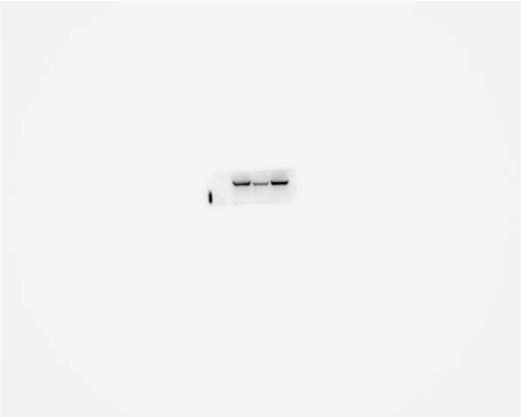

AURKAIP1

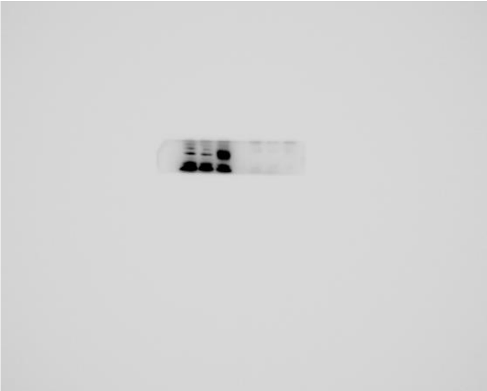

GAPDH

Figure 6

Figure 6E (MDA-MB-231)

β-catenin

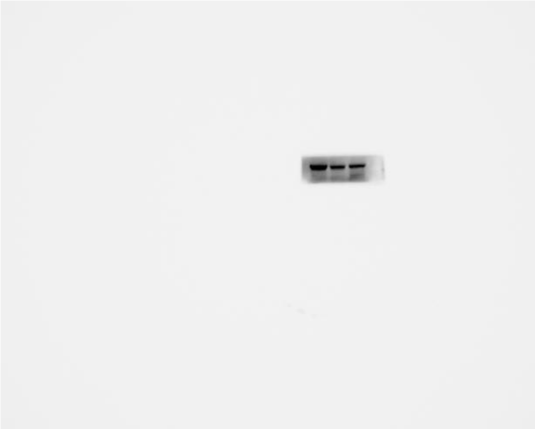

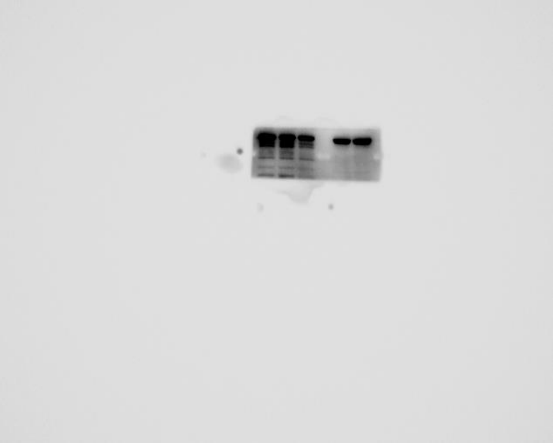

CyclinD1

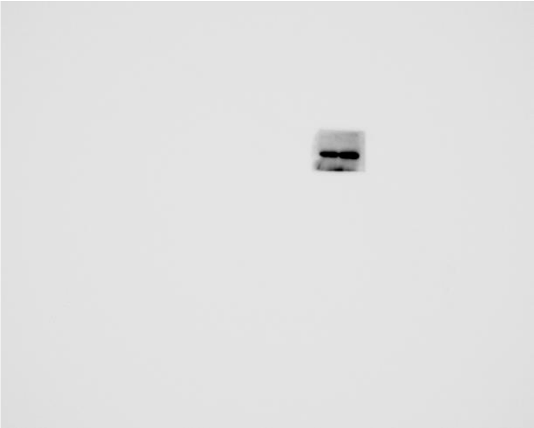

c-Myc

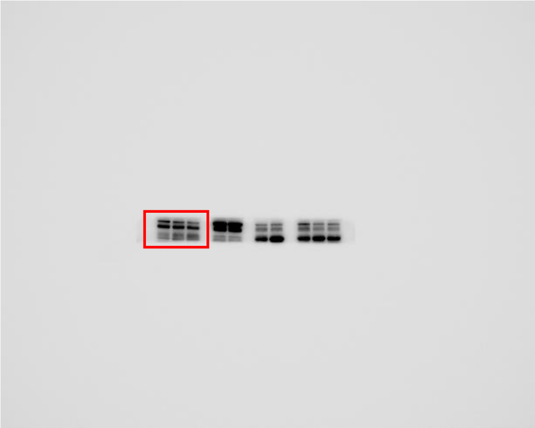

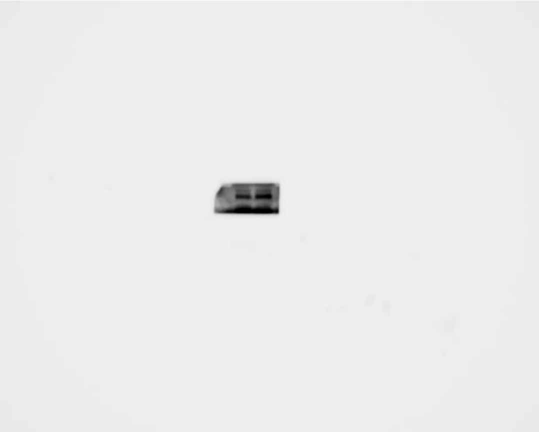

Met

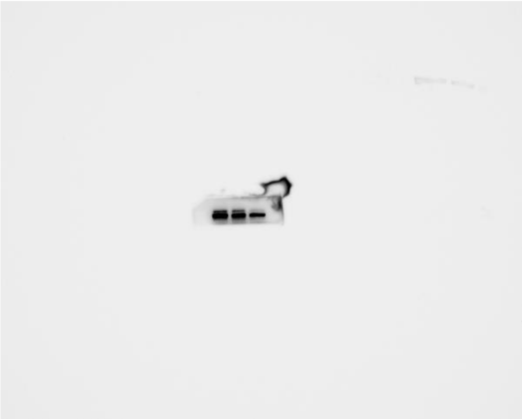

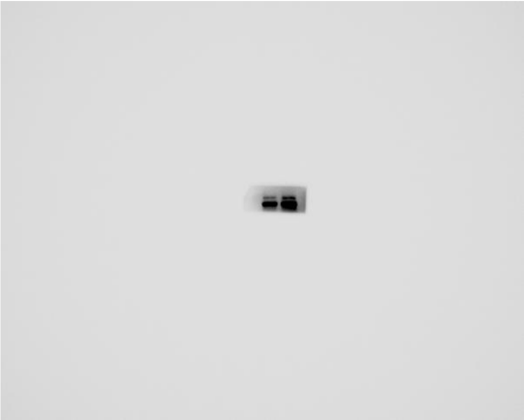

GAPDH

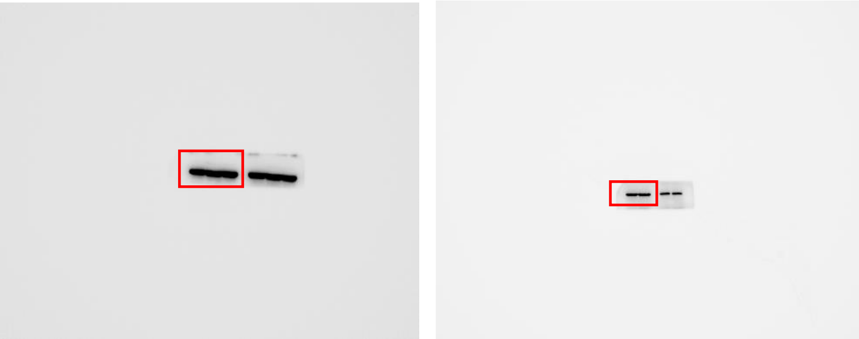

Figure 6E (BT 549)

β-catenin

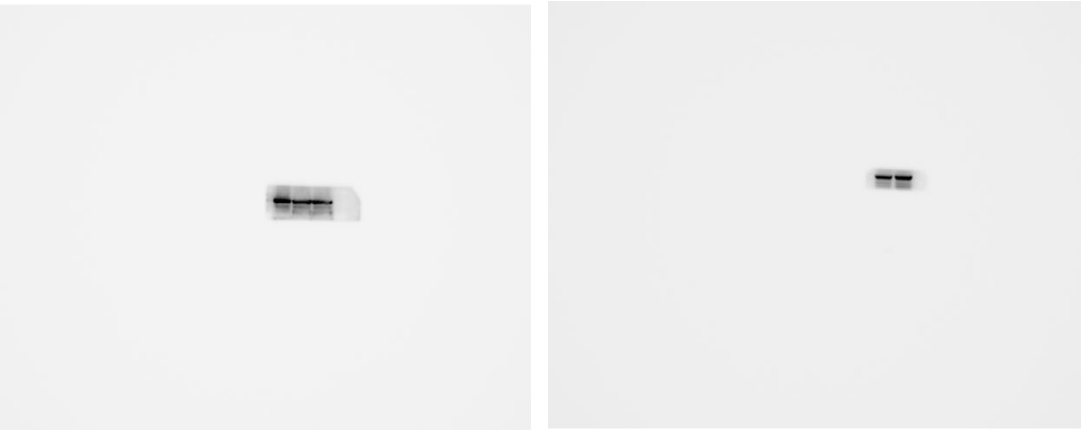

CyclinD1

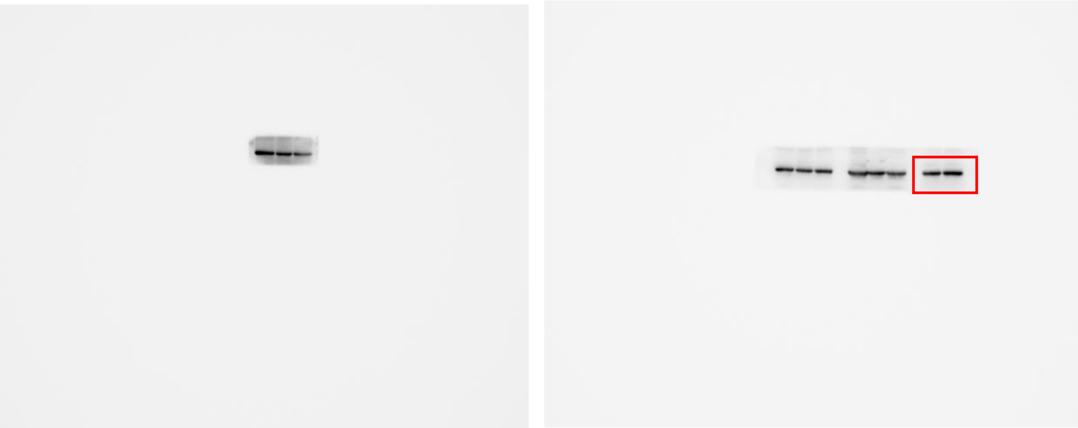

c-Myc

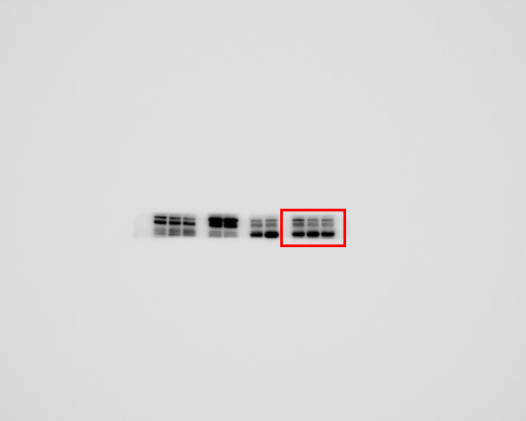

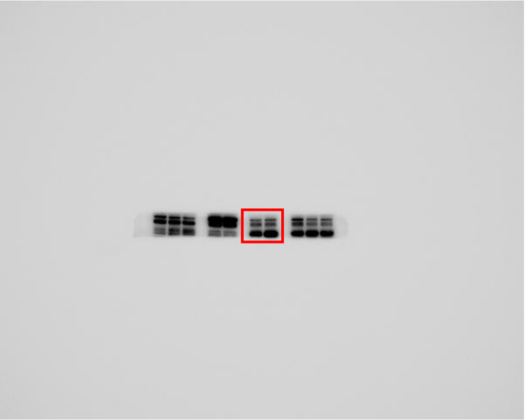

Met

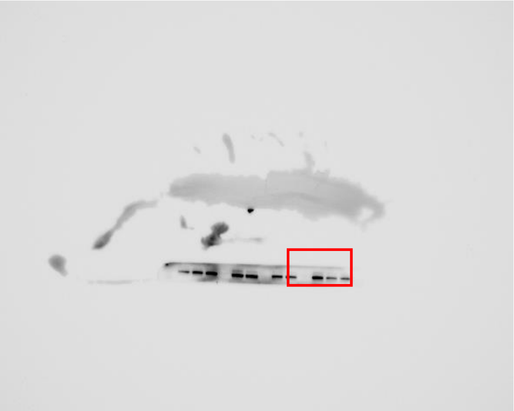

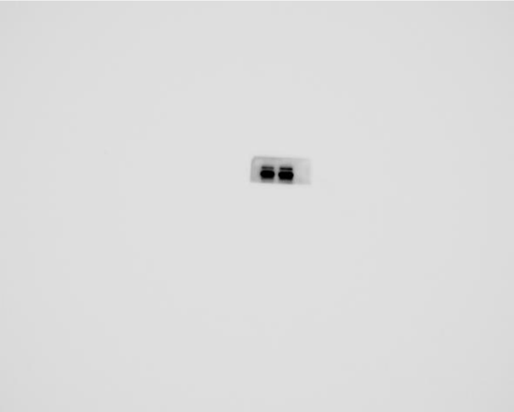

GAPDH

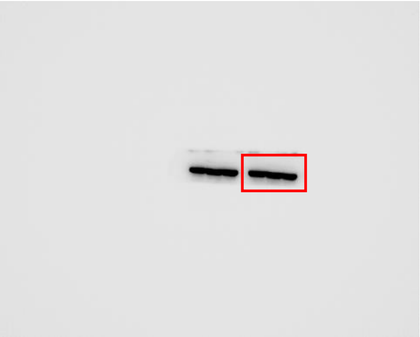

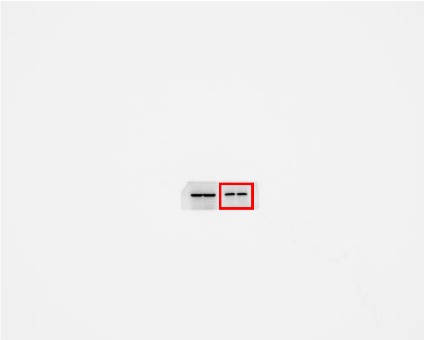

Figure 6J

Figure 6J (MDA-MB-231)

β-catenin

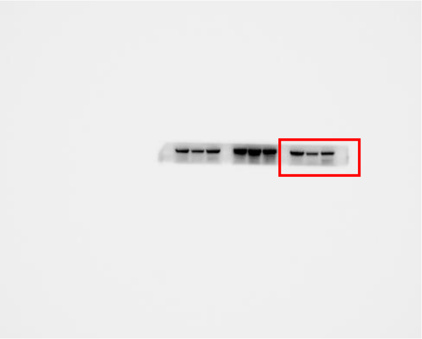

CyclinD1

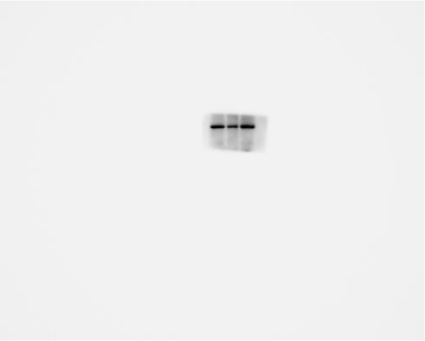

c-Myc

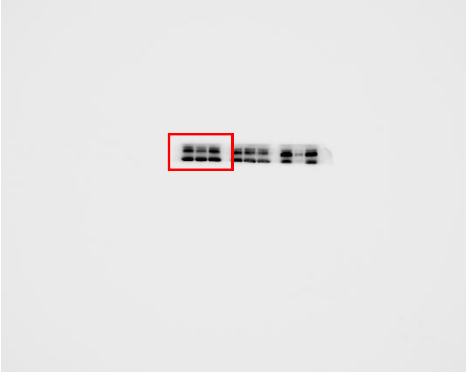

Met

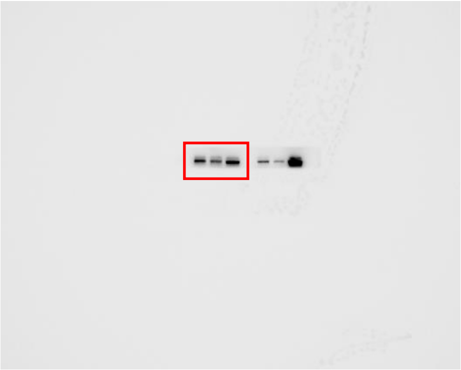

DDX5

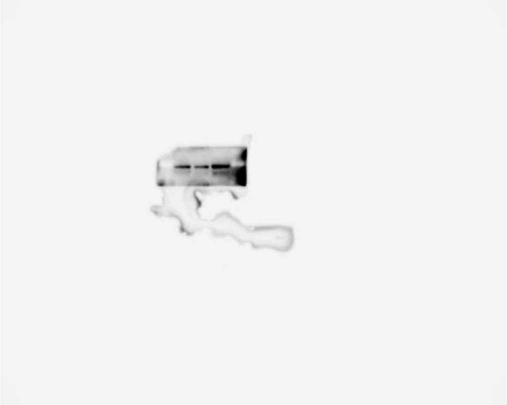

GAPDH

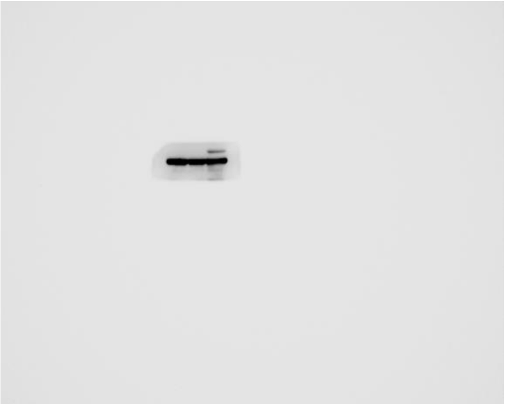

Figure 6J (BT 549)

β-catenin

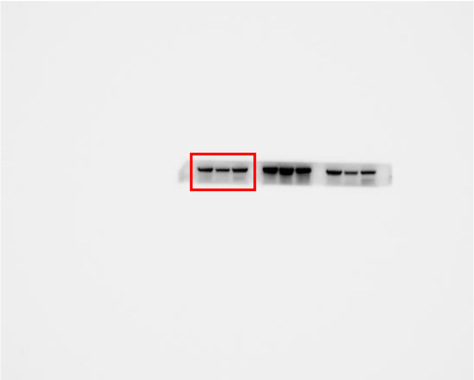

CyclinD1

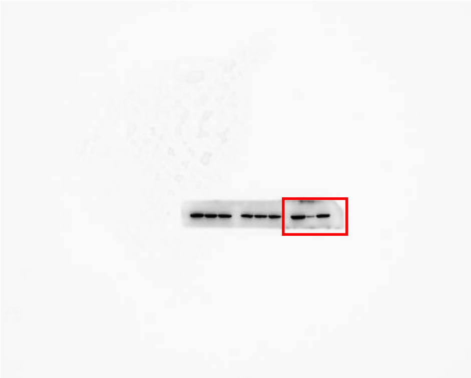

c-Myc

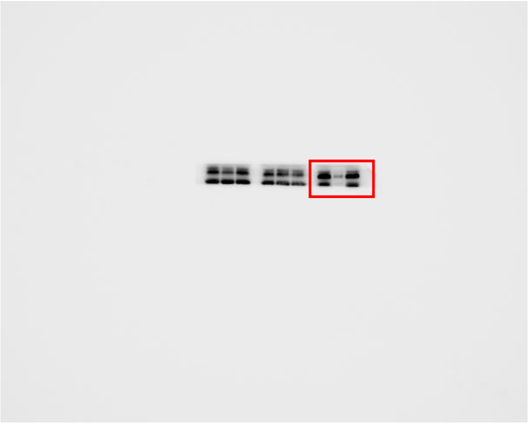

Met

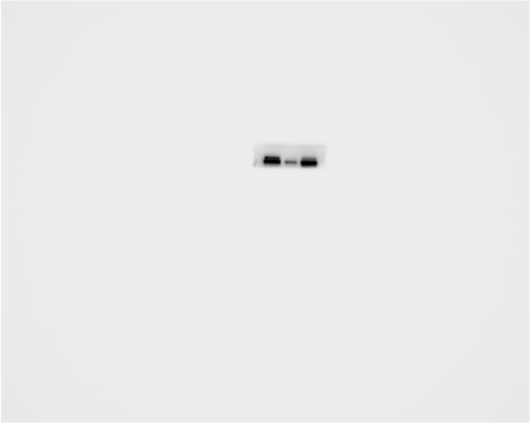

DDX5

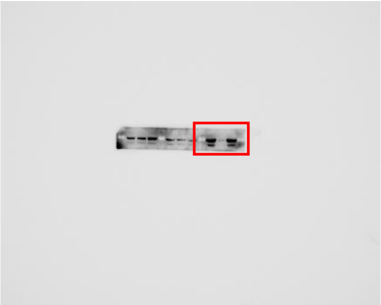

GAPDH

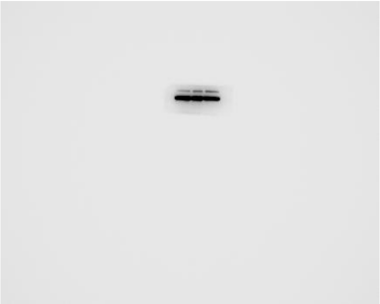

Figure 6M

Figure 6M (MDA-MB-231)

AURKAIP1

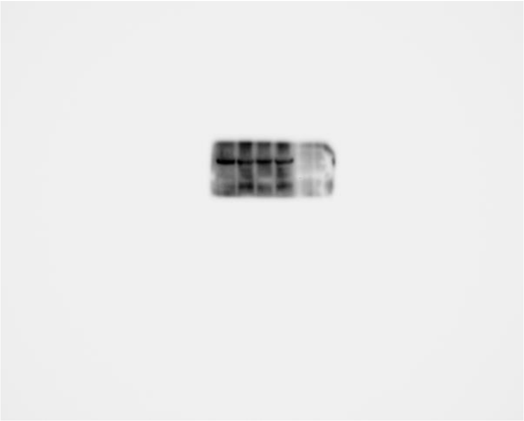

DDX5

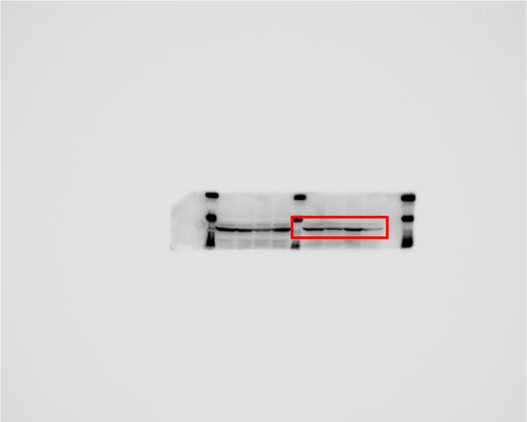

β-catenin

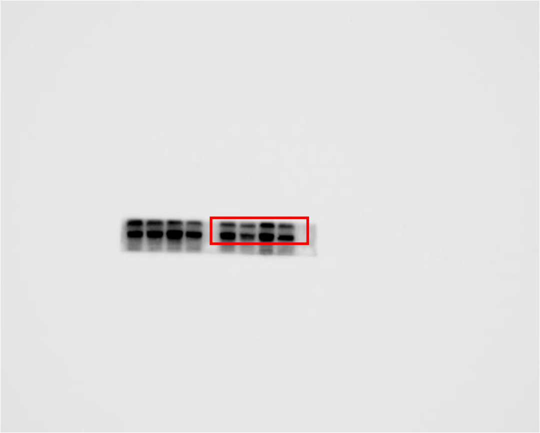

GAPDH

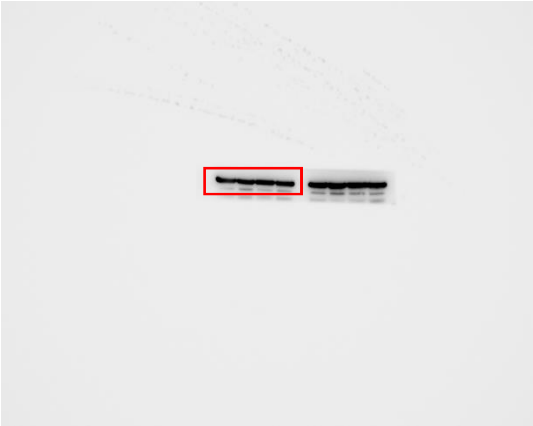

Figure 6M (BT 549)

AURKAIP1

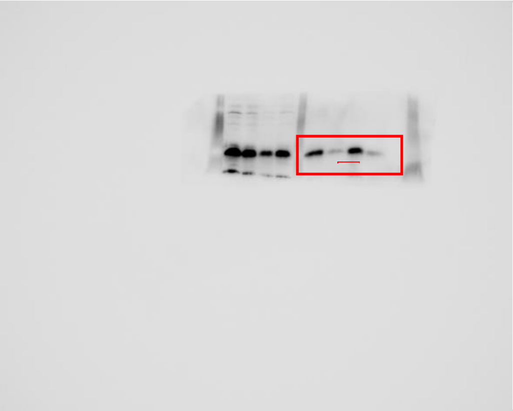

DDX5

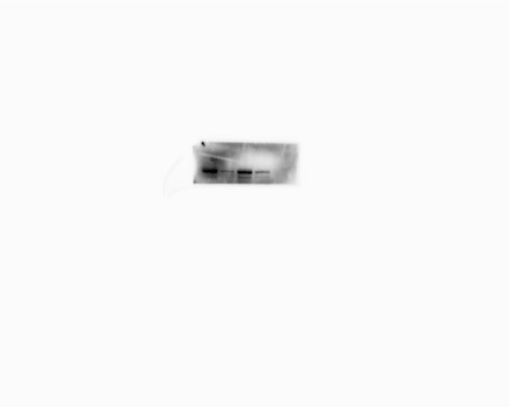

β-catenin

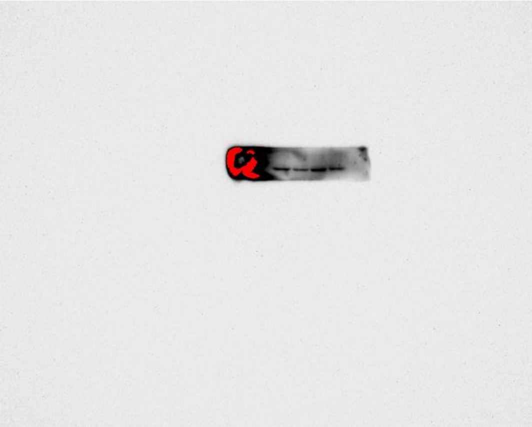

GAPDH

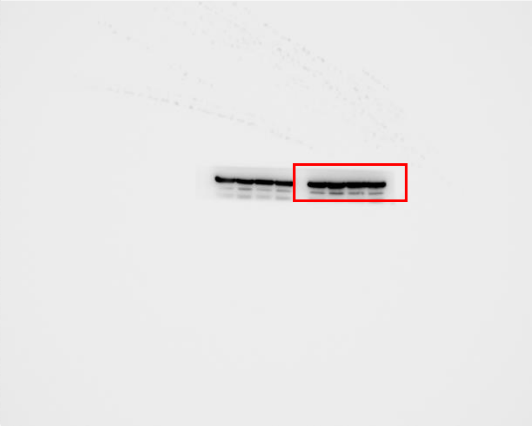

Supplement: Supplementary file 1 — Original data file [file 41419_2023_6115_MOESM1_ESM.pdf]
